# The contributing role of physical education in youth’s daily physical activity and sedentary behavior

**DOI:** 10.1186/1471-2458-14-110

**Published:** 2014-02-04

**Authors:** Senlin Chen, Youngwon Kim, Zan Gao

**Affiliations:** 1 Department of Kinesiology, Iowa State University, Ames, IA 50011, USA; 2 School of Kinesiology, University of Minnesota, Minneapolis, MN 55414, USA

**Keywords:** Childhood obesity, Gender differences, Health, Moderate-to-vigorous physical activity

## Abstract

**Background:**

School physical education (PE) is considered as an effective channel for youth to accumulate moderate-to-vigorous physical activity (MVPA) and reduce sedentary time. The purpose of this study was to determine the contributing role of PE in daily MVPA and sedentary time among youth.

**Methods:**

The study recruited 67 sixth grade children (29 boys; Mean age = 11.75) from two suburban schools at a U.S. Midwest state, 48 of whom contributed ≥10 hours of physical activity (PA) data per day were included for analysis. An objective monitoring tool (i.e., Sensewear armband monitor) was used to capture the participants’ MVPA and sedentary time for 7–14 days. Pearson product–moment correlation analysis (*r*), multi-level regression analyses, and analysis of variance were conducted for data analysis.

**Results:**

MVPA and sedentary time in PE showed significant positive associations with daily MVPA and sedentary time, respectively (*r* = 0.35, *p* < 0.01; *r* = 0.55, *p* < 0.01). Regression analyses revealed that one minute increase in MVPA and sedentary behavior in PE was associated with 2.04 minutes and 5.30 minutes increases in daily MVPA and sedentary behavior, respectively, after controlling for sex and BMI. The participants demonstrated a significantly higher level of MVPA (*p* = .05) but similar sedentary time (*p* = 0.61) on PE days than on non-PE days. Boys had significantly more daily MVPA (p < .01) and less sedentary time (p < .01) than girls; while higher BMI was associated with more sedentary time (p < .01).

**Conclusions:**

PE displayed a positive contribution to increasing daily MVPA and decreasing daily sedentary time among youth. Active participation in PE classes increases the chance to be more active and less sedentary beyond PE among youth.

## Background

The obesity epidemic among children and adolescents has become a major public health concern [1]. Childhood overweight and obesity may lead to a series of chronic diseases that burden modern societies [2,3]. Participating in moderate-to-vigorous physical activity (MVPA) regularly plays a key role in the prevention and control of childhood obesity, as MVPA is positively associated with significant caloric expenditure as well as a variety of health benefits [3]. Research shows that only 42% of children and 8% of adolescents in the U.S. participate in at least 60-minute of MVPA on a daily basis [4]. Not surprisingly, children’s MVPA declines as they grow older, and this trend needs to be reversed through concerted efforts [5,6]. Meanwhile, an average youth spent 3.6 to 8.1 hours being sedentary per day [7]. Prolonged duration of sedentary behavior such as television viewing is related to a variety of health risks [8,9]. As schools can reach out to 95% of children in the U.S., school physical education (PE) is considered a conventional channel to promote MVPA [10-19] and reduce sedentariness. Nevertheless, the role of PE in public health is still under debate due to the diversified natures and lesson foci of different PE curricula across the nation and the world. Further, in a review Pate et al. reported that there were a limited number of studies addressing the public health effects of PE, and these studies did not confirm significant health benefits derived from PE [20]. The national recommendation for engaging in MVPA during PE clarified that a minimum 50% of PE class time should be devoted to MVPA [21,22], but this recommendation has rarely been achieved in numerous PE programs [23,24]. However, this does not suggest PE contributes little to youth’s daily MVPA. On the contrary, in a recent systematic review of 85 published articles, Bassett et al. pointed out that mandatory PE contributed 23 minutes of MVPA per day, the largest contributor compared to classroom activity break, after-school activity program, recess, and others [25]. Given the small proportion of adolescents (8%) who met the recommended 60 minutes of MVPA per day, providing adolescents with regular PE may substantively increase the likelihood for them to meet the recommendations [18].

Engaging in longer duration of MVPA may be associated with the reduction of sedentary time [26]. However, to our knowledge, rarely has research been carried out to objectively quantify MVPA and sedentary time in PE in a single study. Most previous studies [10-19] merely examined the MVPA time acquired from PE classes and did not quantify the proportion of MVPA in PE over a full day or week through continuous monitoring. Therefore, the purpose of this study was to determine the contributing role of PE on daily MVPA and sedentary time in youth. Based on previous studies, it was hypothesized that PE would contribute significant amount of MVPA time but limited sedentary time on a daily basis (due to the disproportion of PE time over the large amount of daily sedentary time).

## Methods

### Participants and setting

All students in the 11 sixth grade classes of two public middle schools in the state of Iowa were invited to participate in the study. As participation was voluntary and required parental consent, a total of 29 boys and 38 girls (age: Mean/SD = 11.75/.50) who returned written parental consent forms constituted the research sample. The sample represented the demographic characteristics of the two schools and typical Iowan middle schools in terms of ethnicity and weight status. It included predominantly Caucasian youth (n = 56, 83.6%), with BMI ranging from 14.99 to 31.68 (Mean/SD = 20.57/3.71), 32.8% being overweight or obese (i.e., 85%ile BMI for 12 old youth = 21.11 [27]). The study was approved by the Iowa State University *institutional review board*.

The middle schools are located in the suburban areas of two small towns in central Iowa. Each school had ample facilities and equipment for PE. Classes were offered two or three times per week on a rotating schedule. The length of the classes ranged from 20 minutes (two hour early release days) to 43 minutes (regular days). The class size ranged from 22 to 39 students. The PE lessons were taught by two certified teachers with 6–9 years of teaching experiences. Both teachers primarily used the direct instruction teaching style [28] to teach PE. At the time of data collection, one school was teaching fitness oriented activities, while the other school was teaching dance. A typical PE day at the first school was sequenced as instant activity, a series of fitness activities with or without equipment, and then closure. The PE teacher posted instant activities to a white board located in the gym before class, then explained and demonstrated the activities in the beginning of class. At the other school, the students were learning a choreographed dance at the time of data collection. The classes began with attendance check, jogging around the gym, dance, and closure. The teacher broke down the dance and taught it step by step. He demonstrated each move and danced with the whole class.

### Instrument

PA level was measured by the Sensewear armband monitor (i.e., armband model: MF-SW; BodyMedia®, 2009; Pittsburg, PA). Each participant was asked to wear the monitor for 7 or 14 days (including at least 2 weekend days and 5 week days). The monitor is a non-invasive, wireless multi-sensor monitor attached to the middle point of the left triceps using an adjustable strap. It relies on several heat-detecting sensors (i.e., heat flux, galvanic skin response, skin temperature, near body temperature, and motion being determined by a tri-axial accelerometer) in addition to a tri-axial accelerometer to measure PA intensity, time, steps, energy expenditure, and other movement-related outcomes. The monitor was set at 1-minute epoch. The proprietary algorithms programmed for the monitor take into account user’s personal information (i.e., age, gender, weight, height, handedness, and smoking status) to determine the MET levels. MVPA and sedentary time (minutes) were obtained to reflect participants’ behaviors over the week as well as in PE classes. One day of measurement was automatically counted from 00:00 to 11:59 pm. As per suggestions from previous research, the cut-off thresholds for MVPA and sedentary behavior were set up as ≥ 4.0 METs and < 1.5METs, respectively, due to the behavioral patterns of youth [29-32]. The monitor is user-friendly and has shown outstanding criterion validity for assessing free-living PA among youth [33].

### Procedures

Data collection occurred in the same season of two years, March and April of 2012 and 2013. The participants in 2012 wore the armband monitors for one week; while those in 2013 wore them for two non-consecutive weeks with an at least one week interval between the two measurement sessions. Despite the uneven durations of the wearing time, this nature allowed more PE classes to be included for data analysis, which increased the statistical power. Furthermore, it did not compromise data quality as our pilot data ensured that participants could demonstrate similar compliance levels in two non-consecutive weeks. The data collection procedures were conducted as follows. First, the participants were instructed on how to use the armband monitors. A researcher gathered the participants and demonstrated the proper way to put on and take off the device. Questions were immediately addressed. A safety guide was distributed and explained to ensure safe experience and credible data. In the middle of the week, a researcher retrieved each participant’s monitor data using a synchronized display device. The retrieved information was shared with the participant as informational feedback. At the end of the week, the armband monitors were collected and data were processed.

### Data analyses

Pearson product–moment correlation was conducted to examine the correlations among MVPA time in PE, sedentary time in PE, daily MVPA time, and daily sedentary time. Two separate multi-level regression models were used to determine the contribution of PE to daily MVPA/sedentary time after controlling for the nesting feature of the current study: days nested within participants and participants nested within trials (either 1 week or 2 weeks). In the regression models, daily MVPA (or daily sedentary time) was specified as the outcome variable, and MVPA time in PE (or sedentary time in PE) along with BMI and gender was specified as the predictors. Shapiro-Wilk test [34] was performed to test for normality of residuals and heteroscedasticity-consistent standard errors [35] were calculated to correct for heterogeneous residuals of the regression models. Cook’s D was used to identify and remove outliers for the regression analyses for MVPA (i.e. 3 participants removed) and sedentary time (i.e. 5 participants removed). To investigate whether MVPA and sedentary behavior would differ between PE days and non-PE days, two separate analysis of variance (ANOVA) were conducted using daily MVPA time and daily sedentary time as dependent variables and day (1 = PE days, 0 = non-PE days) as the independent variable. All statistical analyses were performed with the use of STATA SE/ version 12 (Stata Corp LP., College Station, TX) and significance level was set at α = .05.

## Results

The 67 participants wore the armband monitor for 7 to 14 days, resulting in 534 observations for analyzing MVPA and sedentary time. The weekly level of compliance to use the monitor was between 86.3% and 87.4%. As the study was conducted among youth in free-living environments, this compliance level was deemed high, which ensured the trustworthiness of the recorded data for MVPA and sedentary time. A minimum of 10 hours wearing time per day was required for inclusion [4]. This screening procedure enabled us to remove data outliers caused by inadequate use of the monitor.

Table 1 shows the descriptive results of the daily MVPA time and sedentary time on PE and non-PE days. Since PE classes were canceled on certain days as per the school calendars, only 48 of the 67 participants who took at least one PE class were included to analyze the contributions of MVPA and sedentary time from PE. These 48 participants’ PE-based data yielded to a total of 77 data points for subsequent analyses. The average time spent in MVPA during PE was 15.9 (13.3) minutes and the average PE class duration was 41.5 minutes (2.8). The MVPA time in PE appeared to be substantial, but still less than 50% of the class time (i.e., 20.75 minutes). On average, the participants performed for nearly 2.5 hours of MVPA and 6 hours of sedentary behaviors per day. The contribution of daily MVPA from PE was considerable (nearly 10%), but the accumulation of daily sedentary time from PE was minimal (i.e. 2%). Furthermore, because PE was only offered every other day (i.e., 2–3 times per week depending on the rotation), the contributions of MVPA and sedentary time from PE on a weekly basis were further marginal (1.6% and .3%, respectively). In addition, less than half of the participants performed MVPA for a minimum 50% of the PE class time. Compared to girls, boys engaged in longer duration of MVPA (42.3% vs. 35.4%) and boys were more likely to meet the recommended level of MVPA during PE class (46.9% vs. 37.8%).

**Table 1:** Moderate-to-Vigorous Physical Activity (MVPA) and Sedentary Time in Physical Education (PE), and over the week

**Variable**	**Number of observed data points**	**M**	**SD**
PE time (minute)	77	41.5	2.8
MVPA time in PE (minute)	77	15.9	13.3
Sedentary time in PE (minute)	77	7.6	11.7
% time in MVPA in PE	77	38.4%	32.0%
% time in MVPA in PE (girls)	45	35.4%	28.4%
% time in MVPA in PE (boys)	32	42.3%	36.4%
Total MVPA time/day (minute)	534	151.6	103.7
Total sedentary time/day (minute)	534	383.4	170.7
MVPA contribution from PE/day (%)	77	9.5	8.0
MVPA contribution from PE/week (%)	385	2.2	1.5
Sedentary time accumulation from PE/day (%)	77	1.6	2.3
Sedentary time accumulation from PE/week (%)	385	0.3	0.5

**Figure 1:**
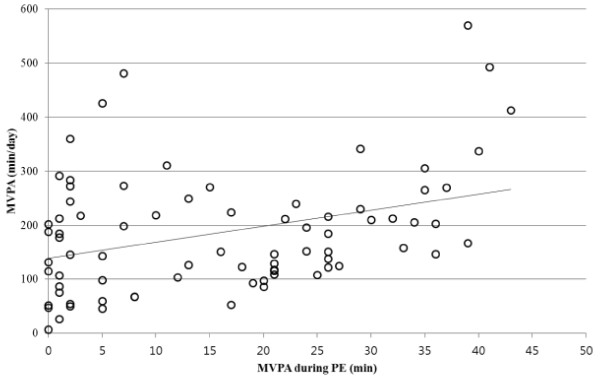
The correlation between daily MVPA time and MVPA time in PE.

**Figure 2:**
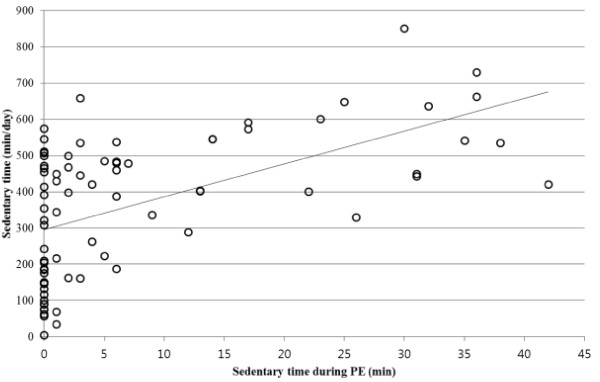
The correlation between daily sedentary time and sedentary time in PE.

Correlation analysis revealed youth’s MVPA time in PE was positively associated with daily MVPA time (*r* = .35, *p* < .01), and sedentary time in PE was positively associated with daily sedentary time (*r* = .55, *p* < .01). MVPA time and sedentary time canceled out each other to some extent, showing a negative correlation pattern (*r* = −.55 for PE and *r* = −.47 for daily, *p* < .01). The associations between daily MVPA time and MVPA time in PE, and between daily sedentary time and sedentary time in PE are presented in Figures 1 and 2, respectively. Subsequent regression analyses, after controlling for sex and BMI, confirmed youth’s MVPA time and sedentary time in PE positively predicted daily MVPA time (−20.39 + 2.04*MVPA time in PE +99.52*Sex + 1.70*BMI, *p* < .01 for MVPA time in PE and sex as predictors) and daily sedentary time (291.36 + 5.30*Sedentary time in PE-174.35*Sex + 14.76*BMI; *p* < .01 for all three predictors), respectively (Girls = 1, Boys = 2). The Shapiro-Wilk test demonstrated that the residuals from both the regression models (i.e. MVPA and sedentary time) were normally distributed (p-values > .05).

**Figure 3:**
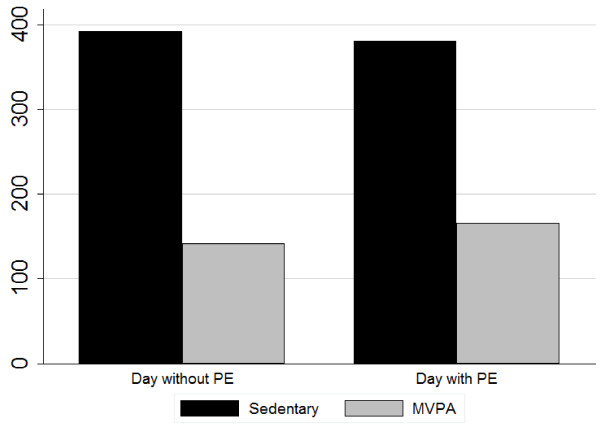
Comparison of MVPA and sedentary time on days with and without PE.

ANOVA tested whether MVPA and sedentary time differed between PE days and non-PE days. It was found that the PE days demonstrated more daily MVPA time (i.e. marginally significant) than the non-PE days (*F*_1,506_ = 3.76, *p* = 0.05). The sedentary time was similar on PE days and non-PE days (*p* = 0.61). The results are illustrated in Figure 3.

## Discussion

This study was intended to examine the contributing role of PE in increasing MVPA and reducing sedentary time among youth. A key strength of this study was that MVPA and sedentary behavior were objectively and concurrently measured using a Sensewear armband monitor. The monitor was found to be consistently used (~87% of the time). Overall, the participants demonstrated approximately 2.5 hours of MVPA per day, 10% of which were accumulated from PE. They participated in MVPA for 38% of the class time in PE, 42% of whom met the recommended 50% of class time in MVPA. Nevertheless, the contribution of PE to daily MVPA was statistically significant, with one minute increment of MVPA in PE predicting over two minutes (i.e. 2.04 minutes) increment of daily MVPA. Further, the daily MVPA level on PE days was found to be higher than that on non-PE days. The above findings verify previous evidence that PE is an important avenue for youth to acquire MVPA [10-19,26], despite the moderate amount of MVPA time received from PE due to constrained instructional time [23,24]. The two PE programs focused in this study offered fitness-oriented activities and dance, which were typical content conveyed in middle school PE. These classes seemed unable to actively engage students for 50% of the class time [15,16]. However, it is important to note that besides offering MVPA, PE as a school subject is entitled to obtain educational objectives (e.g., knowledge and skill acquisition, enjoyable experience) [24,36]. It is equally important to physically engage students and to educate them so that they could gain knowledge and skills. In light of this dilemma, we suggest practitioners seek a common ground where students could find their PE experiences most beneficial [37]. In this present study, the students’ MVPA in PE actually predicted their daily MVPA. The evidence indicates that promotion of MVPA should take concerted efforts of PE teachers, classroom teachers (e.g., classroom-based PA), school administrators (e.g., recess PA), parents (e.g., active transportation), and community leaders (e.g., after-school sports club) [25,26]. Acquiring MVPA from these additional opportunities elsewhere would reduce the public health burden of PE teachers and free them to teach students the competence (e.g., knowledge and skills) and motivation (e.g., intrinsic motivation, goal orientations, etc.) that are essential for sustained long-term engagement of MVPA.

While we applaud PE for making significant contribution to youth’s daily MVPA, this study also found that the sedentary time accumulated in PE was predictive of daily sedentary time. Admittedly, the participants were sedentary for only 7.6(11.7) minutes in PE in the context of 383.4 (170.7) minutes of sedentary time per day and PE days did not significantly reduce the total amount of sedentary time (p = .61). However, one minute increase in sedentary time during PE was associated with 5.3 minutes of increase in daily sedentary time after controlling for sex and BMI. These findings caution that despite the short duration of sedentary time in PE, any additional minutes of sedentary behavior in PE may have a large impact on youth’s daily behavioral pattern. While in reality some sedentary behaviors such as taking class, doing homework, and reading are inevitable, special attention and efforts must be given to reducing recreational sedentary behaviors including television viewing as they are detrimental and pose health risks to youth [8,9]. Therefore, a “health-optimizing” PE [23] should not only stress the promotion of MVPA and the attainment of other educational objectives, it also needs to be alert about and minimize students’ possibility of being sedentary in class. This finding is partially supported by previous research indicating youth could demonstrate high levels of both MVPA and sedentary behavior [38].

An ancillary finding of this study is that girls were less physically active but more sedentary than boys in PE and beyond. Girls were active for 35% of the class time (compared to 42% among boys) and 62% of girls (compared to 53% of boys) did not meet the recommendation (i.e., being active for 50% of PE time). Further, girls demonstrated significantly more sedentary but less active time on a daily basis. Previous research revealed that compared to boys, girls tend to participate in less volitional MVPA and PE is a more important source from which they acquire active time for reaping health benefits [39]. The finding from this present study suggests the need to emphasize girls’ PA participation in conventional PE programs. As a major source of MVPA, PE should strive to protect and entice active in-class participation of girls [40,41]. Last but not the least, participants with higher BMI demonstrated higher sedentariness. While this finding is not surprising [42], it points out that youth as young as 11–12 year old have already shown some behavior patterns (i.e.., prone to sedentary behaviors), which, if habitualized and not intervened in time, would chronically lead to overweight or obesity.

### Potential strength and limitations

A major strength of this study lies in the concurrent research focus on youth MVPA and sedentary behavior. Most previous research studies [10-19] only studied MVPA due to its recognized benefits for health, while youth’s sedentary behavior in free-living settings were often less emphasized. Pate et al. [7] identified sedentary behavior as a construct independent of MVPA in youth, negatively associated with children’s health. More research evidence is needed to investigate both MVPA and sedentary behavior in PE and other school programs among youth.

The findings need to be interpreted with caution due to several limitations embedded in the present study. First, this study was conducted with a small sample constituted primarily by ethnically homogenous individuals (i.e., Caucasians) who were recruited on a voluntary basis. The seemingly higher MVPA level than the norm (1.5-2 hours of MVPA per day for 11–12 year olds) [5] suggest that these participants had higher motivation in PE and/or PA experiences. Although the statistical analyses did not violate the underlying assumptions, the selection bias on sample and lack of diversity limit the generalizability of the findings and our ability to control for other demographic (e.g., social economic status) or contextual variables (e.g., school, class, teacher, etc.). Future research with a larger and more diverse sample is warranted. Also, the observed high MVPA level might have also reflected an overestimation of the Sensewear armband monitor. The existing validation evidence that supports the accuracy of the armband monitor in assessing free-living PA among youth is only available for an older model of the monitor (i.e., Sensewear Pro2) [33]. While the newer model (i.e., MF-SW) is expected to be more accurate, empirical research has not yet explored it. Second, the study did not focus on MVPA and sedentary behavior in other school-based programs such as recess and after-school programs. While this study found that PE is an important avenue to acquire MVPA and reduce sedentariness, future research should examine other school programs to better understand youth’s behavior patterns. Third, the research findings were not based on an experimental research design. Readers should be cautious that the regression results must not be interpreted as causalities between the variables.

## Conclusion

PE contributed significant amount of time to daily MVPA. Daily MVPA time was greater on PE days than non-PE days, but daily sedentary time was comparable between PE days and non-PE days. Boys were more physically active than girls. The findings of this study signal an important public health message in that PE is important in accumulating MVPA in youth’s daily lives. Future research and practice should attach importance to promoting girls’ MVPA in PE as well as in other school-based programs.

## Abbreviations

MVPA: Moderate-to vigorous physical activity; PA: Physical activity; PE: Physical education.

## Competing interests

The authors declare that they have no competing interests.

## Authors’ contributions

SC conceived, designed, and carried out the study, prepared the manuscript for submission. YK analyzed the data and helped to draft the manuscript. ZG helped to draft the manuscript. All authors read and approved the final manuscript.

## Pre-publication history

The pre-publication history for this paper can be accessed here:

http://www.biomedcentral.com/1471-2458/14/110/prepub
